# Intraspinal Lipomas Without Associated Spinal Dysraphism

**DOI:** 10.5812/ircmj.11423

**Published:** 2014-05-05

**Authors:** Erhan Arslan, Kayhan Kuzeyli, Elif Acar Arslan

**Affiliations:** 1 Department of Neurosurgery, School of Medicine, Giresun University, Giresun, Turkey; 2 Department of Neurosurgery, School of Medicine, Karadeniz Technical University, Trabzon, Turkey; 3 Department of Pediatric Neurology, School of Medicine, Hacettepe University, Ankara, Turkey

**Keywords:** Lipoma, Spinal Cord, Spinal Dysraphism, Diagnostic Techniques, Surgical

## Abstract

**Introduction:**

The aim of this study was to report surgical strategies and clinical outcomes for thoraco-lumbar intradural lipomas. Intraspinal lipomas are rare congenital histologically benign neoplasms, which account for less than 1% of all spinal cord tumors. These tumors are most frequently found in the lumbosacral area as components of a dysraphic state, however, intramedullary lipomas are not associated with spina bifida or cutaneous malformations and have only been described as isolated cases among spinal lipomas, where the thoracolumbar region is rarely affected.

**Case Presentation:**

Three patients with thoracolumbar intradural lipomas were admitted to our clinic at different points of time. Partial resections and debulking of the tumors were achieved with the guidance of an operating microscope. We performed laminectomies or laminoplasties, for tumor resections.

**Discussion:**

Postoperatively, the patients demonstrated significant clinical improvements. In this manuscript we presented our surgical experiences for intraspinal lipomas.

## 1. Introduction

Intramedullary lipomas, not associated with spina bifida or cutaneous malformations, have only been described as isolated cases (1-4). The extent of the tumor removal in these cases does not influence long-term results; total excision of the lipomatous lesion is difficult and may not be necessary to reduce the symptoms (3-9). The main purpose of performing the surgery in these cases is to decompress the adjacent neural structures. The goal of this study is to determine how to decide which surgical intervention is proper for symptomatic patients with intraspinal lipomas.

## 2. Case Presentation

### 2.1. Case 1

**Figure 1.**
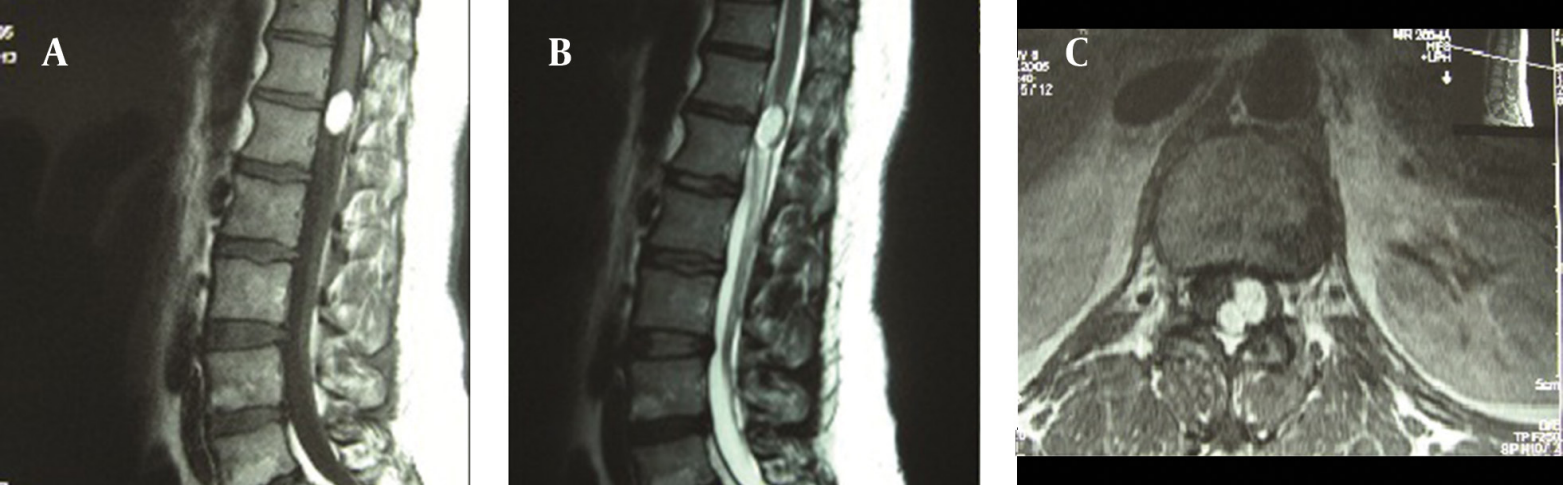
MR Images of Case 1. A pre-contrast sagittal T1W image demonstrating a homogenous well-defined hyperintense intradural spinal cord mass, between Th12 and L1 (A). A T2W sagittal image showing a hyperintense lesion. But the mass lesion is less intense compared to the T1W images (B). An axial T1W image demonstrating the bilobulated mass. The spinal cord is displaced anteriorly by the lesion (C).

A 43-year-old female was admitted in January 2006, with a history of 10 months of lumbago. She had experienced six months of pain and numbness and two months of motor weakness in the left leg. The symptoms had progressed rapidly in the last two months. The neurological examinations showed motor weakness in her lower extremities, predominantly on the left side on tibialis anterior and also crural weakness. She had sensory impairment below the L1 dermatome of the left leg and hypoactive deep tendon reflexes in the lower extremities. She had no urinary incontinence. Rectal tone and sensation were both normal. Routine laboratory studies yielded normal values. Plain radiographs showed no evidence of bone destruction. Magnetic resonance imaging (MRI) revealed a 10 × 14 × 20 mm intradural mass lesion, between Th12 and L1. The lesion was hyperintense on T1-weighted (W) images and less intense on T2-weighted images, indicating the presence of fat tissue (Figure 1A, 1B). The mass appeared posterior to the spinal cord and was displacing the cord anteriorly (Figure 1C). The MRI features led to a straightforward diagnosis of intradural lipoma.

Due to the progressive neurological symptoms, surgery was recommended. The patient underwent a Th12 to L1 laminectomy. Almost no epidural fat was detected at these levels. The dura matter was tight and appeared to be under significant pressure. Following the opening of the dura matter, a typical extramedullary lipoma was apparent. Normal spinal cord was identified above and below the subpial yellow fatty tumor, displacing the spinal cord anteriorly and to the right. The nerve roots were involved bilaterally. The mass was at the extramedullary location but the lack of a cleavage plane was the main difficulty in performing the resection. As a result, extensive debulking of the tumor was achieved with the guidance of an operating microscope and the whole procedure was done using microsurgical instruments only.

Histological examination confirmed the tumor was a lipoma. The postoperative course was uneventful and the patient was discharged on day 10 post-surgery, with improved walking ability. After a 6 year follow-up course, the patient’s walking ability had improved significantly.

### 2.2. Case 2

**Figure 2.**
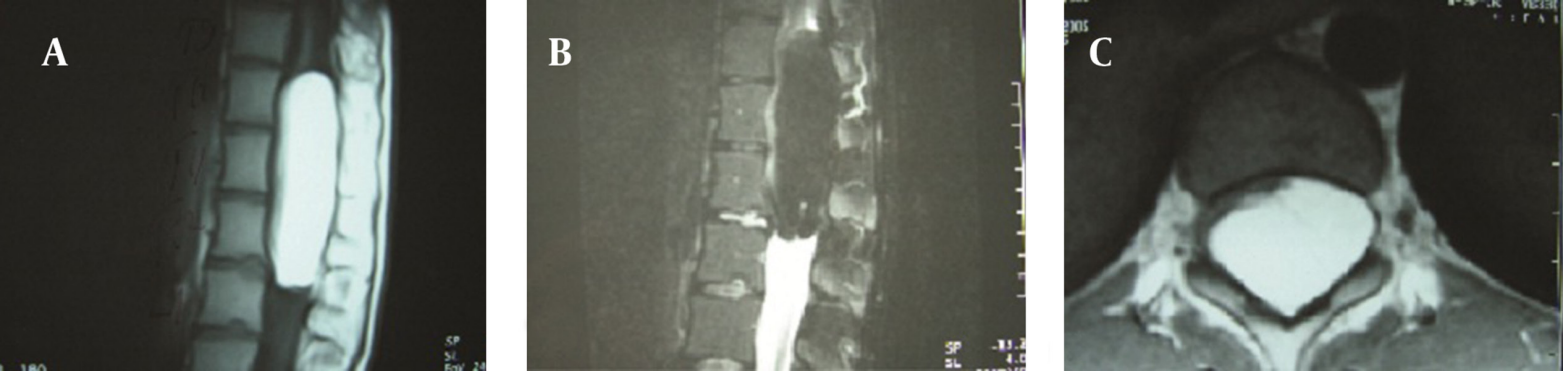
MR Images of Case 2. A pre-contrast T1-weighed sagittal MRI, showing a high signal intensity tumor from Th10 to L1 level (A). The signal intensity dropped dramatically with fat saturation technique, confirming fat as its main component (B). Axial T1W image demonstrates the mass located in an intramedullary position (C).

A 20-year-old female was admitted to our department in September 2005, with a 6-month history of back pain. She had become aware of difficulty in walking and numbness of her legs for four months and the symptoms had progressed rapidly during the last five months. Neurological examination showed dominantly left-sided sensory loss and motor weakness below L1. Sensation to light touch was diminished below L1 and the deep reflexes in the lower extremities were hypoactive. Sagittal MRI showed a tumor mass from the Th10 to the L1 level (Figure 2A, 2B). The mass filled the spinal canal. It appeared to be located in a juxtamedullary position, posterolateral to the spinal cord and was displacing the cord anteriorly and to the right. There was no clear demarcation between the spinal cord and the mass lesion (Figure 2C). The provisional diagnosis of an intramedullary lipoma was made. During the operation, the soft elastic tissue bled moderately, containing all the nerve roots in these levels. Approximately 20% of the tumor was resected. The dura matter was then closed and the Th10–L1 laminae were fixed in the expanded position to increase the canal cross-section. As a result of the extended laminoplasty, spinal stability was sealed for the patient to resume an active life. We chose laminoplasty because the patients were young and it was possible to resect only a small part of the tumor. Also, the tumor was located in the thoracolumbar region (Th10-L1), a dynamic part of spine. Through performing the laminoplasty for extending the spinal canal diameter, spinal stability was preserved. Postoperatively, the patient showed immediate improvements of motor power. When being discharged, the patient was able to walk without assistance. Following a 6.5 year follow-up no walking disability was observed.

### 2.3. Case 3

An 18-year pregnant woman, was admitted to the hospital in 1997 with progressive weakness in her legs and bladder dysfunction for the previous 2 months. Sensation to light touch loss and severe weakness below Th11 was detected, with hypoactive deep reflexes. MRI showed an intradural tumor mass, displacing the cord anteriorly from the Th4 to the Th10 level. The provisional diagnosis was an intradural lipoma. The patient underwent a Th4 to Th10 laminectomy, during which about 40% of the tumor, which was firmly adherent to spinal cord, was resected. During the operation an ultrasonic aspirator and operating microscope were used for debulking intramedullary lipoma. Although the patient was young, we chose laminectomy, since the thoracic localized masses (Th4-Th10), rarely develop post-laminectomy spinal instability and affect the patient’s active life. Spinal canal diameter was extended sufficiently.

Motor function improved slightly after the operation. Histopathological diagnosis was lipoma. During the postoperative period, the patient’s condition improved rapidly and she was able to walk better on the fourth postoperative day. After a 15 year follow-up, improved walking ability was observed.

## 3. Discussion

Gowers was the first to describe an intraspinal lipoma in 1876 (10). The more common lipomatous malformations, associated with spinal dysraphism have been extensively reviewed in the literature (5, 6), however, intramedullary lipomas not associated with spina bifida or cutaneous malformation have only been described as isolated cases (1-4). The classical location of these tumors is intradural and they may be intramedullary, extramedullary or a combination of the two. The first presented case was an extramedullary tumor but in case 2 and 3, the masses were at the intramedullary location.

These lesions are likely to develop at the cervicothoracic, thoracic or cauda equina regions, but may also involve the entire length of the cord (10) and extend to the foramen magnum (7, 11-14). The following theories have been put forward to explain the lipoma pathogenesis (15):

(a) metaplasia of the adipose tissue in the pia-arachnoid membrane,
(b) a developmental error, in which lipoma can arise from inclusions of embryonic rests, within the meninges during the formation of the neural tube and
(c) proliferation of fat cells, which are occasionally found in the pia matter.

Experiences with imaging of the isolated lipomas are very limited, due to the scarcity of these tumors. The low density of the fat tissue produces the pathognomic appearance of lipoma on computed tomography. The characteristic MRI findings of lipomatous tissue are relatively high signals on T1W images and relatively low signals on T2W images. Although lipomas may tend to adhere to the surface of the cord, a chemical shift artifact may erroneously suggest the presence of a thick fibrous tissue plane between the cord and the mass (16).

Although it is benign, surgical removal of an intradural spinal lipoma is virtually impossible due to the extensive adhesions to surrounding neural tissues (17). Trying to remove all of the mass can lead to parenchymal injury, sometimes resulting in impaired neurological functions, immediately after the surgery. Recent technical advances, like the ultrasonic aspirator, surgical laser and operating microscope have significantly improved surgical outcomes for patients with intraspinal lipomas. The ultrasonic aspirator can be used for debulking intramedullary lipomas (3, 9). However, due to the significant vibration of the ultrasonic aspirator, it can be hazardous for treating lipomas that are adherent to the nerves and spinal cord. Fujiwara reported good postoperative outcomes in three out of four cases, following partial removal of lipomas, using an ultrasonic aspirator (1). Laser surgery may be effective for gentle debulking of these tumors (9, 12, 18-20). Xu reported marked improvement or recovery in 45 out of 58 cases after laser treatment (20). Both Heary and Crols concurred that partial removal of the lipoma does not impair a patient’s postoperative neurological status (5, 21).

Many authors claim that the extent of tumor removal in these cases does not influence long-term results and that total excision of the lipomatous lesion is difficult and may not be necessary to reduce symptoms (3-9). The main purpose of surgery in these cases is to decompress the adjacent neural structures.

Debulking of intraspinal lipomas should be performed if it is possible for surgeon to easily dissect it from surrounding neural tissues. If extensive adhesions to neural tissues were observed on surgery, partial resection of intraspinal lipoma should be performed.

In most cases additional decompression of neural structures by laminectomy or laminoplasty is also necessary to relieve the neurological symptoms. Laminectomies should be preferred after partial resection of the lipomas located at the thoracic levels, which are the more stable segments of the vertebral colon and if the patient is not young. If additional need for spinal instrumentation occurs after performing laminectomies in young-aged patients, laminoplasties should be chosen.

In our cases, the tumors were intradural and firmly blended with the cord, with the spinal roots embedded in the adipose tissue. During the surgery, we encountered difficulties posed by the absence of a cleavage plane and adhesion of tumor tissue to neural structures. We decided to extensively debulk the patient’s lipoma, using an operating microscope and were able to avoid damage to adjacent neural tissue. Adding decompression to partial tumor resection in case 2 and case 3, significant improvements were observed in postoperative neurological status. In case 1, an intramural extramedullary lipoma was localized on the conus medullaris. Although the lipoma was extramedullary, some parts of the mass adhered to the spinal cord. For this reason, the only option left was extensive debulking. Th12-L1 laminectomies were performed for spinal cord decompression in this case. We preferred laminectomies because the patient was middle-aged (43). Preserving the facet joints, when doing laminectomies, there was no need for spinal instrumentation. In case 2, intramedullary lipoma located at Th10-L1 levels was adherent to the spinal cord and spinal roots were embedded in the lesion. We performed partial tumor resection (20 % of the mass) and Th10-L1 laminoplasties. By doing laminoplasties, we achieved spinal canal diameter expansion. We chose to perform laminoplasty to ensure a longer active life expectation, due to the patient’s very young age (20) and the probability of developing spinal instability in the future due to the dynamic structure of the thoracolumbar region. In case 3, lipoma was an intramedullary lesion, located at Th4-Th10 levels. The tumor did not allow more extensive resection. Partial tumor resection and decompression was performed by doing thoracic laminectomies. We did not prefer to choose laminoplasty, although the patient was very young (18) or did not perform spinal stabilization after laminectomies for the preservation of facet joints, due to the stable structure of the thoracal segment of the spine. We summarized the cases and performed surgical intervention at Table 1. According to our surgical experiences, the factors determining the surgical strategy for this type of intraspinal lipomas are the patient’s age, localization of intraspinal lipoma in spine; being intra- or extramedullary, the amount of lipoma adhesion to the spinal cord and the relationship between the lipoma and nerve roots.

Indications for surgery and the surgical strategies for treatment of spinal intradural lipomas are still discussed controversially.

**Table 1.** Cases With Intraspinal Lipomas Operated With Different Surgical Techniques

Case	Age and Sex	Lipoma Localization	Performed Surgical Technique
**1**	43 F	Th12-L1 extramedullary	Extensive debulking + laminectomy + decompression
**2**	20 F	Th10-L1 intramedullary	Partial resection (20%) + laminoplasty + decompression
**3**	18 F	T4-T10 intramedullary	Partial resection (40%) + laminectomy + decompression
